# Consanguineous Marriage and Its Association With Genetic Disorders in Saudi Arabia: A Review

**DOI:** 10.7759/cureus.53888

**Published:** 2024-02-09

**Authors:** Abdullah M Khayat, Balsam Ghazi Alshareef, Sara F Alharbi, Mohammed Mansour AlZahrani, Bashaer Abdulwahab Alshangity, Noha Farouk Tashkandi

**Affiliations:** 1 Pediatrics, College of Medicine, Taif University, Taif, SAU; 2 Biotechnology, College of Science, Taif University, Taif, SAU; 3 Family Medicine, College of Medicine, Taif University, Taif, SAU; 4 Medical Research, College of Medicine, King Saud bin Abdulaziz University for Health Sciences, Riyadh, SAU

**Keywords:** consanguineous marriage, congenital heart diseases (chds), renal diseases, saudi arabia, genetic disorders

## Abstract

Consanguineous marriages, where spouses are related by blood, have been a longstanding practice in human history. The primary medical concern with consanguineous marriages is the increased risk of genetic disorders. When closely related individuals reproduce, there is a higher probability that both parents carry the same genetic mutation. In Arab countries, especially Saudi Arabia, the rate of consanguineous marriage is high compared with Western European and Asian countries. This high rate is directly proportionate with elevated risk of genetic disorders, including congenital heart diseases, renal diseases, and rare blood disorders. Additionally, it was noted that the rate of negative postnatal outcomes is higher in consanguineous marriages compared with the general population. These observations indicate the necessity of tackling this area and highlighting the consequences of this practice. In this review, we aim to discuss the current evidence regarding the association between consanguineous marriages and genetic disorders in Saudi Arabia.

## Introduction and background

Consanguinity, the practice of marriage between individuals who are closely related genetically, has been a subject of medical and genetic interest due to its implications for offspring [1]. This practice, prevalent in some cultures and communities, raises significant concerns regarding the increased risk of genetic disorders in children born from such unions [2]. The primary medical concern with consanguineous marriages is the heightened risk of genetic disorders. When closely related individuals reproduce, there is a higher probability that both parents carry the same genetic mutation. This situation increases the likelihood of recessive genetic disorders in their children. Recessive disorders, such as cystic fibrosis, thalassemia, and Tay-Sachs disease, occur only if a child inherits two copies of the mutant gene, one from each parent [3]. In consanguineous unions, the chances of both parents carrying the same recessive gene are significantly higher compared to non-consanguineous marriages. As a result, the incidence of autosomal recessive disorders is more frequent in populations where consanguinity is commonly practiced [4].

Moreover, consanguinity can lead to an increase in the expression of deleterious genes, leading to a reduction in overall genetic diversity within a family or community. This reduced genetic diversity can have broader implications beyond single-gene disorders [5]. It may affect complex traits and the overall health of the population, potentially leading to reduced immunity and increased susceptibility to infectious diseases. Additionally, the accumulation of deleterious mutations over generations can result in a higher prevalence of multifactorial diseases, such as heart disease and diabetes, which are influenced by both genetic and environmental factors [6]. Therefore, the implications of consanguineous marriages extend beyond the immediate risk of single-gene disorders, impacting the broader health and genetic resilience of communities where it is practiced. In this review, we aimed to discuss the current evidence regarding the association between consanguineous marriages and genetic disorders in Saudi Arabia.

## Review

Prevalence of consanguinity

Consanguineous marriages, where spouses are related by blood, have been a longstanding practice in human history. Currently, around 20% of the global population resides in areas where such marriages are preferred [7]. The prevalence of consanguineous unions varies across different societies, influenced by factors like religion, culture, and geographical location. In Western and European nations, the occurrence of CM is less than 0.5%, while in India, the prevalence stands at 9.9% [3]. On the other hand, consanguinity is particularly prevalent in many Arab nations, with rates ranging from 20 to 50% of all marriages. In these regions, first-cousin marriages are especially common, averaging around 20-30% [7].

The preference for marrying relatives in Arab communities is largely driven by socio-cultural reasons. These include preserving family structure and assets, facilitating marriage arrangements, fostering harmonious relations with in-laws, and economic benefits related to dowries [8]. While consanguineous marriages are often perceived as more stable compared to those between non-relatives, there is a lack of comparative studies on divorce rates in Arab societies. It is commonly believed that in marital disputes, the husband's family tends to support a consanguineous wife, as she is seen as part of the extended family. This support extends to caring for children with disabilities, with more family members involved in their care. Interestingly, the practice of consanguinity is not exclusive to Muslim communities in the Arab world; Christian communities in Lebanon, Jordan, and Palestine also engage in it, albeit to a lesser extent [9-12].

**Figure 1:**
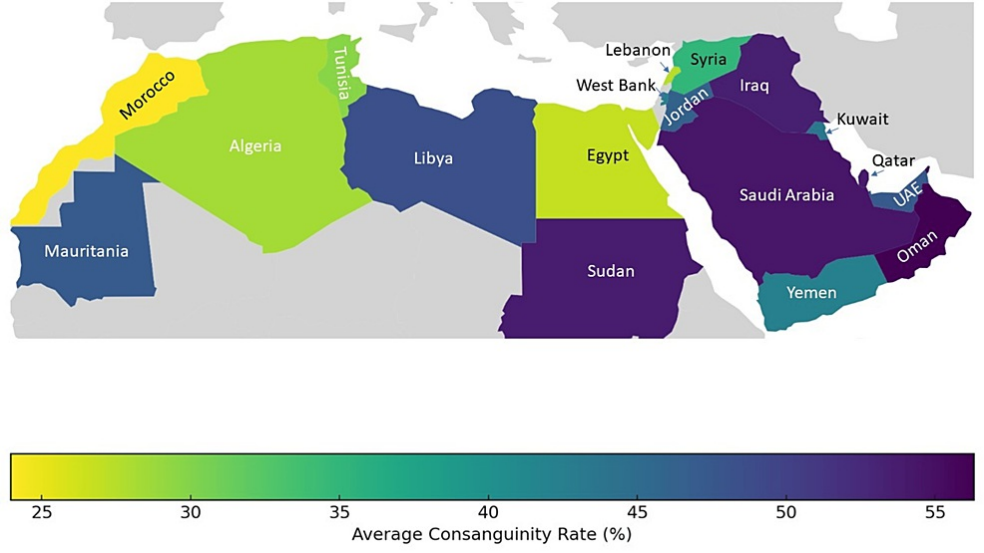
Consanguinity rates in the Middle East and North Africa Image credits: Noha Farouk Tashkandi

The rates of consanguineous marriages show significant variation both between and within Arab countries (Figure 1). Sometimes, marriages between third cousins or more distant relatives are included in consanguinity statistics, which can affect overall rates but not significantly alter the average inbreeding coefficient. For comparing consanguinity rates across populations, two key measures are used: the mean inbreeding coefficient (F) and the incidence of first-cousin marriages. The tradition of consanguinity in Arab societies is long-standing, and the cumulative estimate of F might surpass the value calculated for a single generation [13].

In some Arab populations, a decline in consanguineous marriages has been observed, as seen in Jordan [14], Lebanon [12], Bahrain [15], and Palestinians [16-18]. This decrease could be attributed to several factors, including higher education levels among women, reduced fertility rates leading to fewer eligible relatives for marriage, increased urbanization, and improved family economic status. Additionally, as infectious diseases become less of a threat, there is growing concern over genetic disorders. Typically, the highest rates of consanguineous marriages are found in rural areas and among the poorest and least educated segments of society [13]. Urban areas tend to have lower consanguinity rates compared to rural ones, as evidenced by data from Algeria (10%-15%), Egypt (8.3%-17.2%), and Jordan (29.8%-37.9%) [11,19,20]. In Jordan, for instance, the consanguinity rate is inversely related to the educational level of the female partner; university-educated women are less likely to marry first cousins compared to their male counterparts [11]. This trend of lower consanguinity rates among educated women is also observed in Yemen [21] and Tunisia [22].

Despite these changes, consanguineous marriages are still favored in many Arab countries, including Qatar [23], Yemen [21], the UAE [24], and Tlemcen in Algeria [20]. In Morocco, studies show conflicting trends, with one indicating an increase in consanguinity [25] and another a decrease [26]. The persistence of consanguineous marriages in some regions can be attributed to the perceived social benefits outweighing the disadvantages [27]. These marriages are valued for promoting family stability, simplifying premarital financial negotiations, ensuring compatibility between spouses and family members, reducing the risk of undisclosed financial and health issues, and preserving family land [8,28,29]. A study in Jordan found that consanguinity could be protective against violence during pregnancy among 390 women attending reproductive health clinics [30]. However, caution is advised when interpreting these secular trends, as significant regional and ethnic variations in consanguinity prevalence are common in countries where it is widely practiced [8].

Saudi Arabia ranks among the countries with the highest incidence of consanguineous marriages globally. Various studies have highlighted differing rates influenced by time and location. A 1989 study by Saedi-Wong and Al-Frayh revealed a 54.3% prevalence of such marriages [31]. In 1995, research by el-Hazmi and colleagues indicated an overall prevalence of 57.7%, breaking down to 28.4% for first-cousin marriages, 15.2% for distant relative marriages, and 14.6% for second-cousin marriages. The highest recorded rate was 80.6% in Samtah, while the lowest was around 34% in Abha, located in the South Western province [32]. A 1997 survey involving 2001 married Saudi individuals found that 51.3% (1022 marriages) were consanguineous, with 41.1% of these being second-cousin marriages or closer [33]. Subsequent research in 1998 reported a 52.0% prevalence of consanguineous unions, with first-cousin marriages constituting 39.3% of these [34]. A decade later, in 2007, El-Mouzan et al. reported a slightly higher overall prevalence of 56%, with first-degree cousin marriages (33.6%) being more common than other types (22.4%). This study also noted a higher prevalence in rural areas (59.5%) compared to urban ones (54.7%), with significant regional variations - 67.2% in Madina and 42.1% in Al-Baha [35]. More recent data from 2014 indicated a decline in consanguineous marriages (including first and second cousins) to 37.9%. Recent investigations by Albanghali in Albaha, Saudi Arabia, revealed a consanguinity rate of 40% (N = 302) in marriages. This study highlighted that out of these marriages, first- and second-cousin unions constituted 72% and 28%, respectively. Notably, when comparing the prevalence of consanguineous marriages between the study participants and their parents, a lower rate was observed in the older generation (31%) as opposed to the participants (40%) [36].

Association between consanguinity and genetic diseases

The identification of disease genes has often been successful in populations that are geographically or culturally isolated. This success is largely due to the phenomenon of chromosomes being inherited identically by descent (IBD), where a segment of a chromosome from one parent is identical to the corresponding segment from the other parent [37]. In scenarios where parents share a close genetic relationship, their children are more likely to inherit autosomal recessive disorders. This increased risk stems from the fact that genetic relatedness heightens the probability of disease-causing mutations manifesting in homozygous segments. Consequently, analyzing these homozygous segments in such populations becomes a crucial approach for identifying the specific region of the mutation responsible for the condition.

In autosomal recessive disorders following simple Mendelian inheritance, an affected individual receives the disease-causing IBD allele from both parents. This allele originates from a common ancestor and is passed down through different branches of the family tree, converging in the offspring of a consanguineous marriage (often represented by double lines in a pedigree). Offspring from such unions typically have multiple homozygous chromosomal regions of IBD. However, the region containing the variant responsible for the disease will be common among affected individuals but not homozygous in unaffected ones. Recombination events during meiosis can narrow down these IBD alleles, potentially isolating the disease-causing region to a very small segment.

The detection of homozygosity stretches using genetic markers in inbred or consanguineous pedigrees laid the groundwork for homozygosity mapping in positional cloning [38]. While IBD homozygosity is often the likely cause of a rare autosomal recessive trait in consanguineous families, there's also a risk of compound heterozygote mutations, common in offspring from non-consanguineous unions, which might be overlooked by strict homozygosity mapping [39]. Therefore, analyses should also aim to identify linkage in regions of compound heterozygosity [40].

Advancements in sequencing technologies, like whole exome or genome sequencing, have accelerated gene discovery [41]. However, as more DNA is sequenced, more variants are identified, posing a challenge in distinguishing the disease-causing mutation from other genetic changes. Therefore, it is essential to combine these modern technologies with established mapping techniques like linkage analysis. This integration is key to efficiently focusing the search for the disease-causing allele.

Genetic consequences of consanguinity

Determining the precise health risks associated with consanguineous marriages for their offspring is challenging due to various interfering factors, such as socioeconomic status and the quality of healthcare available. A significant study highlighted an increased risk of 4.4% for pre-reproductive mortality in children born from first-cousin unions [42]. Additionally, other research has indicated an increased risk of congenital anomalies ranging from 0.7% to 3.8% [28]. However, these figures should be approached with caution. They could be affected by a range of variables, including social and economic conditions, demographic factors, variations in reproductive patterns, and differences in morbidity and mortality rates at both early and late stages [43].

Cardiovascular disease

Consanguinity often leads to a higher occurrence of autosomal recessive disorders in children, resulting in elevated morbidity and mortality rates [44,45]. This increase is due to the higher likelihood of individuals carrying multiple abnormal genes meeting and producing offspring with a combination of pathogenic variants. In their research, Monies et al. performed clinical exome sequencing (CES) on more than 2200 Saudi families that had not been previously studied. This led to the identification of 155 genes as potential candidates for recessive diseases within the Saudi demographic [46]. Among the autosomal-recessive variants discovered, 77.2% were unique variants, 98.4% were found in a homozygous state, and 41.3% were considered founder variants [46]. It is theorized that there is a correlation between the degree of homozygosity in the genome and the level of consanguinity [47]. In populations with high rates of consanguinity, the gene pool, including homozygous genes, is likely to contain defective alleles. Furthermore, populations with consanguineous marriages and comparable mutation rates might see a rise in the frequency of allelic and locus heterogeneity. Evidence of this was provided by Kamal et al., who discovered four previously unknown mutations in the ALMS1 gene in Saudi patients suffering from the rare autosomal recessive Alstrom disease. This finding indicates allelic heterogeneity within this inbred group [48].

In a large cohort study involving over 1500 patients, Chehab et al. explored the link between consanguinity and the incidence of congenital heart disease (CHD). The research indicated that among the patients, 19% were offspring of first-degree cousins, 5.9% from second-degree cousins, and 2.1% had parents with other types of consanguineous relationships. On the other hand, the control group, which did not have CHD, showed 14% of children born to first-degree cousins, 5.9% to second-degree cousins, and 3.6% from other forms of consanguineous unions, highlighting a significant difference [49]. Additionally, the study found notable variations in consanguinity rates among different types of CHD. The highest incidences of valvular aortic stenosis (AS), tetralogy of Fallot (TOF), and atrial septum defect (ASD) were observed in children with consanguineous parents, pointing toward a recessive genetic component [49].

The association between consanguinity and CHD is further supported by additional studies [3,50]. Becker et al. conducted a comprehensive study on genetic diseases within the Saudi population, utilizing a random selection of families from all regions of Saudi Arabia [50]. The preliminary findings from the first 1013 Saudi families who underwent testing with gene panels and whole-exome sequencing (WES) revealed a significant variation in diagnostic effectiveness. The most substantial diagnostic results were seen in instances involving multiple congenital malformations in prenatal environments and skeletal dysplasias. Importantly, testing couples who had lost children yielded a notably high diagnostic rate of 83%, including the discovery of new candidate genes. Within this group, autosomal recessive mutations that were pathogenic or likely pathogenic comprised 71% of the cases. Of these, 97% were homozygous mutations, which mirrors the high level of consanguinity (78% among the 482 families who provided information about consanguinity). Founder mutations were responsible for 33% of these recessive positive cases [50].

The role of parental consanguinity in the prevalence of CHD in the Saudi population is well established [3,51]. A study involving 1028 CHD patients from the Congenital Heart Disease Registry at King Faisal Specialist Hospital in Riyadh revealed a significantly higher rate of first-cousin marriages among CHD patients compared to the general population. This study linked first-cousin consanguinity with a higher incidence of pulmonary atresia (PA), pulmonary stenosis (PS), atrioventricular septal defect (AVSD), ventricular septal defect (VSD), and ASD, suggesting that consanguinity may amplify genetic risk factors for certain cardiac defects in a highly inbred population [52].

Recent cross-sectional research indicates that in Saudi Arabia, the incidence of CHD varies from 2.1 to 10.7 cases per 1000 individuals. The most frequently occurring condition is VSD, accounting for 29.5 to 39.5% of these cases. This is followed by ASD, which comprises 8.9% to 18.1% of the cases, and PS, making up 6% to 12.4% of the incidences. The occurrence of CHD in Saudi Arabia has been significantly associated with factors such as Down’s syndrome, consanguinity, and maternal diabetes [51]. Overall, some cohort studies over 25 years have highlighted the importance of consanguinity as a risk factor for CHD in the Saudi population, as summarized in Table 1.

**Table 1:** Saudi studies that reported association between consanguinity and CHD CHD: Congenital heart disease, COA: coarctation of the aorta, TA: truncus arteriosus, TOF: tetralogy of Fallot, PDA: patent ductus arteriosus, AS: aortic stenosis, PS: pulmonary stenosis, PA: pulmonary atresia, AVSD: atrioventricular septal defect, ASD: atrial septal defect, VSD: ventricular septal defect

Study	City	Setting	Data collection year	CHD sample	Conclusion
Becker et al., 1997 [50]	Riyadh	CHD Registry	1998	949	The occurrence of CHD was notably higher in children from first-cousin marriages, at 41.6%, compared to 28.4% in the broader population.
Hamamy 2012 [3]	Nation-wide	Household	2004–2005	11,554	Among the respondents, 56% indicated that CHD was the sole condition linked to first cousin consanguinity.
Shaheen et al., 2015 [53]	Dhahran	Hospital	1996–2000	37 families	In these families, 23 marriages (62%) were consanguineous. The occurrence of dilated cardiomyopathy was notably higher in consanguineous unions, with 26 cases, compared to just 2 cases in non-consanguineous marriages.
Dasouki et al., 2020 [54]	Riyadh	CHD Registry	1998	891	The study revealed that the rate of consanguinity in the sample group was considerably higher at 40.4%, compared to 28.4% in the general population. Certain types of CHD, including PS, PA, AVSD, ASD, and VSD, showed a significant correlation with consanguinity. However, this association was not observed in cases of PDA, COA, AS, TA, or TOF.

The hereditary nature of CHDs is supported by their recurrence in families and their link with inherited microdeletion syndromes [55]. A considerable number of CHD cases are associated with chromosomal anomalies, particularly in syndromic cases that include multiple growth problems, developmental delays, and organ deformities. Chromosomal aneuploidies were among the earliest recognized genetic causes of CHD [56]. These affect roughly 50% of children with trisomy 21 (occurring in one out of every 600 births) [57], 20-50% of individuals with Turner syndrome (one in every 2500 female births) [58], and nearly all children with trisomy 13 and 18 [59]. Cardiac malformations vary in aneuploidy syndromes, with specific lesions like AVSD in trisomy 21 and coarctation of the aorta (COA) in Turner syndrome, while others like transposition of the great arteries (TGA) are less common [56]. These genotype-phenotype correlations initially suggested that heart malformations result from altered doses of specific genes rather than a broad genomic change [56].

Al-Hassnan et al. conducted a comprehensive array of CGH studies on a Saudi cohort with CHD [60]. They identified cytogenetic imbalances in 17 CHD cases with additional conditions like autism spectrum disorder, intellectual disability, and developmental delays. Notably, a chromosomal deletion on 12p12.1 involving SOX5 was found in a child of consanguineous parents [60]. This supports previous findings linking SOX5 haploinsufficiency to various CHD anomalies [61]. Additional studies are required to delve into the role of clustered CNVs in the context of CHD. In an effort to examine genetic anomalies in Down’s syndrome patients with CHD, Alharbi et al. created a gene panel that targets chromosome 21 and other autosomes. Through this analysis, they pinpointed specific defects in the KCNH2, GUSB, and GATA3 genes unique to these patients [62]. Additionally, splice variants in FLNA were found in isolated Down’s syndrome cases and those with CHD, suggesting a role in DS pathogenesis. Other genetic variations in CEP290, ENG, and MEF2A were also implicated in abnormal cardiac development [62]. Almawazini et al. recently documented a significant occurrence of CNVs in a range of CHDs, particularly in chromosomal areas chr 22q11.23, 8p11.21, 16p11.2, and 17q21.31, within a substantial Saudi cohort. Their functional and network analyses emphasized the importance of several genes, such as NR3C1, KANSL1, PLCB1, and NPHP1, in the development of CHD [63]. These discoveries indicate a clustering of alterations in genes and pathways that are vital for cardiovascular functionality and development.

Blood disorders

Approximately 75% of patients with hemophilia, the most prevalent bleeding disorder, reside in developing countries. Yet, only about 20% of those with common bleeding disorders in these regions are diagnosed [64]. The situation for diagnosing and determining the prevalence of rare bleeding disorders (RBDs) in developing countries is likely not more favorable than that for hemophilia. RBDs, which include genetic deficiencies in coagulation factors I, II, V, VII, XI, X, XIII, combined factor V + VIII deficiency, and multiple vitamin K-dependent factor deficiencies, account for about 3-5% of all inherited bleeding disorders. These disorders are primarily inherited in an autosomal recessive manner. The incidence of homozygous forms of RBDs in the general population ranges from 1 in 500,000 to 1 in 2 billion [65]. Regions with high rates of consanguineous marriages tend to see a greater prevalence of RBDs. Both race and consanguinity have been identified as influencing factors in the prevalence of RBDs [66]. Consanguineous marriages, which are prevalent in roughly 20% of the global population, tend to perpetuate the recessive genetic traits in homozygous states within families [3,67].

Rare bleeding disorders

RBDs are found in the general population at a rate varying from 1 in 500,000 to between 1 and 3 billion [68]. Initially, RBDs appear to have uniform frequencies across different global regions. However, in areas with a high prevalence of consanguineous marriages, the risk of offspring inheriting bleeding disorders is notably elevated, potentially exceeding the frequency of hemophilia B [69]. This has led to a surge in epidemiological, clinical, laboratory, genomic, and therapeutic research, enriching our understanding of RBDs [70]. Despite this, in developing countries where consanguineous marriages are common, access to high-quality healthcare is often limited, making the diagnosis and treatment of RBDs, particularly severe forms, a challenge. Additionally, the economic feasibility of developing treatments for a small patient population is a concern, particularly in the Middle East and parts of Asia, where RBDs are more common. Conditions like vWD type 3 or factor VII deficiency require specialized care in tertiary medical centers, which are often concentrated in the capitals of developing countries. This poses accessibility issues, especially for women with RBDs who need specialized care during pregnancy [71].

The high incidence of consanguineous marriages and large families in Middle Eastern countries increases the likelihood of females with hemophilia. A comprehensive family history, including details of consanguineous unions, is crucial for genetic counseling and guides the scope of laboratory testing. In countries like Pakistan, where consanguineous marriages are prevalent, a significant proportion (89% of 429 patients) of bleeding disorder cases were found in consanguineously married parents, with nearly half (49%) having RBDs [72]. This highlights the need for specific healthcare strategies and possibly legislative measures in regions with high RBD incidence. In Saudi Arabia, there is a lack of evidence regarding the association between RBDs and consanguinity, indicating the need for well-established studies.

von Willebrand disease (vWD)

In a study involving 533 individuals from a Pakistani tribe, 98 (18.4%) were found to have a bleeding disorder, with vWD and platelet disorders being the most common [73]. Many women with vWD often remain undiagnosed for extended periods despite experiencing bleeding episodes since childhood [74]. vWD is classified into three types: 1, 2 (subtypes 2A, 2B, 2N, 2M), and 3. The inheritance of type 2N, type 3, and some cases of type 2A and type 1 is autosomal recessive, while most cases of types 1, 2A, 2B, and 2M follow an autosomal dominant pattern [75]. In the absence of genetic testing, vWD is diagnosed based on clinical and laboratory features. The high prevalence of vWD suggests that women with menorrhagia but without pelvic anomalies should be tested for the disease [76].

In the Middle East, where consanguineous marriages are prevalent, an increased incidence of vWD is anticipated. Due to the minor bleeding tendency associated with some vWD forms, certain subtypes may go undiagnosed. Children born to consanguineously married couples, both of whom have undiagnosed vWD, may have a severe bleeding tendency. Many bleeding disorders, including vWD, are inherited in an autosomal recessive manner. The likelihood of a carrier of a rare bleeding disorder finding a partner with the same genetic defect is low in the general population but significantly higher in consanguineous marriages. Factor XI deficiency and factor VII deficiency, among other RBDs, are more prevalent in the general population [77]. These insights underscore the importance of considering consanguineous marriage as a factor in suspecting RBDs in patients with bleeding disorders.

Platelet disorders

Glanzmann’s thrombasthenia (GT) is a notably rare platelet disorder characterized by a deficiency in platelet aggregation, except when induced by ristocetin. This disorder stems from a mutation in the 5-007ib 3 integrin, leading to dysfunctional platelet aggregation [78]. The severity of bleeding in GT can vary, ranging from minor petechiae to life-threatening hemorrhages. GT is more commonly observed in communities with a high rate of consanguineous marriages. Reports have indicated an increased incidence of GT in children born from consanguineous unions, particularly in regions where marrying close relatives is a cultural norm [75,79]. In 2013, a study identified a severe bleeding disorder in siblings from a consanguineous marriage. This condition was linked to a mutation in the human CalDAG-GEFI gene (RASGRP2), which resulted in the patient's platelets not responding to any aggregating stimuli [80].

Renal diseases

In children, genetic factors are responsible for over 70% of kidney diseases [41]. In Saudi Arabia, where specific epidemiological data is scarce, the high prevalence of consanguinity likely contributes to genetic kidney disorders being the predominant cause [81-84]. The prevalence of autosomal recessive diseases in Saudi Arabia, much higher than in other regions, is largely attributed to consanguinity [85]. Children in Saudi Arabia show a higher incidence of genetic kidney diseases compared to their global counterparts. These include familial nephrotic syndrome, congenital urologic anomalies, renal tubular acidosis, nephrocalcinosis syndrome, familial hypomagnesemia with hypercalciuria, polycystic kidney disease, and familial juvenile nephronophthisis [85]. Comparable patterns are seen in Kuwait [86], Turkey [87], and Lebanon [88], countries where consanguinity is similarly widespread. The prevalent consanguinity in Saudi Arabia has been instrumental in uncovering specific genetic kidney diseases for the first time in the region. One such example is the “marble brain disease,” reported by Ohlsson et al., characterized by osteopetrosis, renal tubular acidosis, and cerebral calcification [89]. This disease, associated with intellectual disability and stunted growth, is linked to a deficiency in the carbonic anhydrase II enzyme [90]. Long-term studies on this disease have also been conducted in Saudi Arabia [91]. Additionally, new syndromes have been identified and reported in the country [92,93]. Recent advancements in genotyping and sequencing technologies have improved the detection of mutations in known disease genes in individuals with inherited kidney diseases [94]. Techniques such as whole-genome single-nucleotide polymorphism analysis combined with exome sequencing on genomic DNA from affected family members are instrumental in identifying genes responsible for kidney diseases with similar histopathological features.

Consanguinity and congenital malformations

Between 3 and 5% of all newborns globally are born with a significant birth defect. A recent March of Dimes report highlighted that birth defect rates in most Arab countries exceed 69.9 per 1000 live births, which is higher than the rates in Europe, North America, and Australia, where it is below 52.1 per 1000 live births [95]. However, the United Arab Emirates and Kuwait recorded lower rates, at 7.92 and 12.5 per 1000 births, respectively [96,97]. In Oman, out of 21,988 births, major malformations were observed in 24.6 per 1000 births [98]. The variation in birth defect rates across different countries and studies could be due to actual differences in populations, varying definitions and methods of identifying birth defects, or differences in the timeframes of the studies. The risk of birth defects in offspring of first-cousin marriages is estimated to be 2-2.5 times higher than in the general population, largely because of autosomal recessive disorders [99-101]. Another estimate suggests a 1.7-2.8% increased risk of congenital defects in children of first cousin unions above the general population risk [102]. However, these estimates require further validation in Arab countries through more controlled, evidence-based, and standardized research.

Studies in Arab countries consistently show higher frequencies of consanguineous marriages among parents of children with congenital malformations compared to the general population. This trend is observed in the UAE [103,104], Kuwait [97], Oman [105,106], Iraq [40,41], Jordan [107,108], Egypt [109], Tunisia [110], and Saudi Arabia [111]. Higher rates of consanguinity were also observed among parents of newborns with congenital hydrocephalus [112] and neural tube defects [113,114]. A correlation between consanguinity and cleft lip and/or palate has also been reported in Saudi Arabia [115].

Consanguinity and postnatal mortality

In regions where consanguineous marriages are prevalent, the impact of consanguinity on mortality rates tends to be lower compared to areas with fewer consanguineous unions [116]. This trend is not unexpected, considering the lack of comprehensive control for factors such as socioeconomic status, maternal education, birth spacing, and the quality of public health services in many studies on consanguinity. A recent large-scale study involving over 600,000 pregnancies and live births revealed that children born to first cousins face a 4.4% higher rate of pre-reproductive mortality than those from non-consanguineous relationships [42]. In Saudi Arabia and Sudan, most research indicates that postnatal mortality rates are higher in children of consanguineous parents compared to those of unrelated parents [117,118]. However, some studies have not observed this increase in postnatal mortality [119]. The elevated postnatal mortality in offspring of consanguineous couples may be attributed to harmful recessive genes and complex genetic factors inherited from common ancestors. Interestingly, the higher number of children typically born to consanguineous couples tends to offset the increased infant mortality rate, often leading to a similar number of surviving children between consanguineous and non-consanguineous families.

Strategies and opportunities

Saudi Arabia's newborn screening (NBS) program, aimed at detecting inherited metabolic diseases (IMD), currently covers only a small fraction of newborns, primarily through select clinical centers or by referring symptomatic infants. The program utilizes tandem mass spectrometry (MS-MS) and dried blood spots (DBS) for screening, a method acknowledged for its accuracy and reliability [120]. It has been found that nearly half of the diseases identified through MS-MS are treatable [121]. However, despite early detection, many conditions lead to considerable morbidity, and the financial burden of ongoing treatment can be overwhelming. Identifying the genetic causes of these disorders and providing comprehensive screening and counseling to reduce the birth rate of children with IMD or other recessive disorders is the ideal strategy.

Mandatory premarital screening for conditions like sickle-cell anemia and β-thalassemia in Saudi Arabia represents a shift toward informed decision-making in marriages, rather than outright prevention of unions at genetic risk. This approach, particularly for hemoglobinopathies and thalassemia, has gained acceptance due to the simplicity and affordability of tests like hemoglobin electrophoresis. However, extending premarital screening to include other prevalent recessive disorders hinges on the development of molecular genetic screening techniques.

The implementation of molecular genetic screening in Saudi Arabia is fraught with challenges, including the diversity of disorders and significant scientific and financial considerations. The high costs and logistical complexities of such screening raise questions about its practicality and extent. Accurate epidemiological data, which are currently limited, would be invaluable in setting screening priorities. One proposed approach is to utilize DBS samples from the NBS program for molecular genetic screening to determine the incidence and geographical distribution of common hereditary diseases. For widespread disorders like hereditary deafness, thalassemia, and hemoglobinopathies, pre-conceptual screening or broad-based premarital could be justified. In contrast, for less common diseases, targeted screening of extended families might be more feasible, as evidenced by β-thalassemia prevention programs in Pakistan and Sardinia [122]. A combination of population-based and inductive screening methods may be necessary in Saudi Arabia, tailored to each specific disease.

Considering the variety of inheritable diseases that could gain from screening and the allelic diversity present in some of these conditions, the expense associated with molecular genetic screening might be viewed as excessively high. To ensure that such screening is cost-effective, it is vital to utilize existing program infrastructures, take advantage of economies of scale, and prioritize the multiplexing of screening tests. Current developments are focused on automated procedures using DBS and whole genome amplification, which utilize the efficiency of the φ29 polymerase and random hexamers to produce significant amounts of DNA from minimal genomic DNA samples [123]. Successful template DNA generation from DBS for real-time PCR-based assays has been achieved. The adoption of these multiplexing technologies is vital for the success of an extensive preventative program in Saudi Arabia.

## Conclusions

In conclusion, this comprehensive review has highlighted the significant impact of consanguineous marriages on the prevalence and spectrum of genetic disorders, particularly in the context of Saudi Arabia. The review underscores the increased risk of autosomal recessive disorders and congenital anomalies among offspring of consanguineous unions, reflecting the genetic implications of such marriages. It also emphasizes the role of consanguinity in the expression of rare genetic diseases and the importance of genetic counseling and community awareness in managing these risks. Overall, the findings from various studies provide critical insights into the genetic consequences of consanguineous marriages, offering valuable guidance for healthcare policy and practice in regions where such marriages are prevalent.
